# Penetrating injury to the chest by an attenuated energy projectile: a case report and literature review of thoracic injuries caused by "less-lethal" munitions

**DOI:** 10.1186/1749-7922-4-26

**Published:** 2009-06-26

**Authors:** Joao Rezende-Neto, Fabriccio DF Silva, Leonardo BO Porto, Luiz C Teixeira, Homer Tien, Sandro B Rizoli

**Affiliations:** 1 Risoleta Tolentino Neves University Hospital Trauma Center – Universidade Federal de Minas Gerais, Belo Horizonte, Brazil; 2 Coordenacao de Aperfeicoamento de Pessoal de Nivel Superior (CAPES), Esplanada dos Ministérios Bloco "L" – Anexo II 2° andar, Brasilia, Brazil; 3 Sunnybrook Health Sciences Centre, University of Toronto, Toronto, Canada

## Abstract

We present the case of a patient who sustained a penetrating injury to the chest caused by an attenuated energy rubber bullet and review the literature on thoracic injuries caused by plastic and rubber "less-lethal" munitions. The patient of this report underwent a right thoracotomy to extract the projectile as well as a wedge resection of the injured lung parenchyma. This case demonstrates that even supposedly safe riot control munition fired at close range, at the torso, can provoke serious injury. Therefore a thorough investigation and close clinical supervision are justified.

## Background

Currently, crowd control is ideally enforced by a trained police force using "less-lethal" tactics and weapons. Previous reports of serious injuries and even deaths, caused by hard rubber bullets, have prompted the development of safer, attenuated energy rounds [1-3]. Examples include the plastic baton rounds and the more recent attenuated energy projectile. These rounds represent safer options than the original rubber bullets and are currently used by many different police forces.

We report a rare case of a penetrating injury to the chest caused by an attenuated energy projectile. We then review the historical development and injury literature surrounding rubber and plastic "less-lethal" impact munitions.

## Case presentation

**Figure 1:**
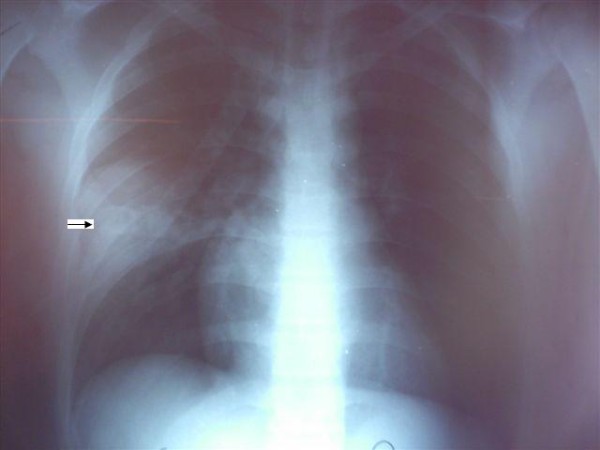
**Admission chest radiography**. Admission chest radiograph shows a radiopaque image within a pulmonary contusion (arrow), and a small pneumothorax on the right hemithorax.

**Figure 2:**
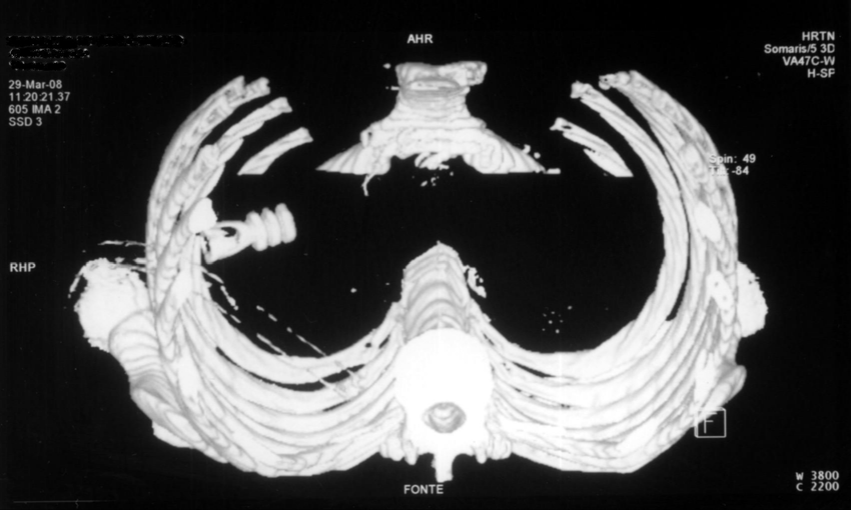
**Admission CT scan of the chest**. CT three-dimensional (3D) image reconstruction of the chest shows an intra-thoracic attenuated energy projectile and a chest thoracostomy tube inside the right hemithorax.

**Figure 3:**
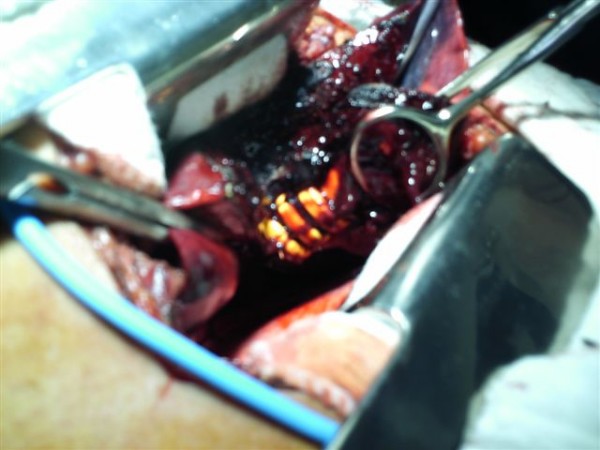
**Intra-operative finding**. Intra-operative photograph depicts the AM-403/P attenuated energy projectile within the lung parenchyma during wedge resection.

A 24-year-old male was shot in the right hemithorax by an attenuated energy projectile (AEP), fired from a 12-gauge shotgun at close range (less than 3 m). He arrived to our Trauma Center approximately 36 hours after the injury. He presented with a fever, had decreased breath sounds on the right side, and his vital signs were stable (pulse was 100, blood pressure was 140/90 mmHg. Physical examination revealed a single skin laceration (2.0 cm) with surrounding contusion at the right mid-axillary line; 4^th ^intercostal space. The admission chest radiograph revealed a small right pneumothorax, pulmonary contusion and radiopaque material within the right middle lobe (Figure 1). A right-sided thoracostomy tube drained minimal air and blood. A computed tomography (CT) scan of the chest demonstrated a foreign body in the right hemithorax with the form of an AM-403/P attenuated energy projectile (Figure 2). Due to septic complications and the size of the foreign body, the patient underwent a right thoracotomy which revealed a 19 g (6.5 × 2.5 cm) projectile within the middle lobe, which was surrounded by an intra-parenchymal hematoma (Figure 3). The projectile and injured parenchyma were removed by wedge resection. The patient had an uneventful hospital stay and was discharged home 5 days later.

## Discussion

"Less-lethal" weapons are explicitly designed and primarily employed to incapacitate personnel, while minimizing fatalities [4]. There are many classes of "less lethal weapons" including conducted electrical weapons (commonly referred to as a TASER), chemical irritants (Pepper spray), and impact munitions. Impact munitions include "bean bag rounds", rubber bullets, plastic baton rounds, and attenuated energy projectile. As our case is an example of a serious injury caused by a rubber bullet, we focused our literature review on chest injuries caused by rubber and plastic "less lethal" munitions from 1972 to 2008 (Table 1).

**Table 1:** Articles published in the English language pertaining to thoracic injuries caused by rubber and plastic "less-lethal" impact munitions (1972–2009)

**Author/Year**	**Bullet Type/Speed/** **Energy**	**Range** **(m)**	**Total Cases/Chest**	**Intra-thoracic Penetration**	**Significant thoracic injuries**	**Outcome**
**Shaw J**. **1972**	Rubber 150 g/ 116.5 m/s/*	27.4	3^	No	Lung contusion (3)	All survived
**Millar R**. **1975**	Rubber 140 g/73 m/s/*	*	90/18	No	Lung contusion(5), pneumothorax(1), rib fracture(2)	All survived
**Sheridan S**. **1983**	Plastic 135 g/*/*	*	147/21	*	*	*
**Rocke L**. **1983**	Plastic/*/*	*	99/10	No	Lung contusion(7), rib fracture(1)	All survived
**Ritchie A. 1990;1992**	Plastic 134.5 g/69.4 m/s	*	123/70	Yes	Lung contusion, hemo-pneumothorax, rib fracture, sternum fracture, myocardial contusion, cardiac tamponade.	5 Deaths
**Walden R**. **1990**	Plastic/*	*	1/1	Yes	Arterial embolization.	Survived
**Missliwetz J. 1991**	Plastic pellets 1 g/302 m/s/ 694J	4.5	4/1	Yes	Soft tissue injury	Survived
**Yellin A**. **1992**	Plastic 8.5 g/*/*	*	26/26^•^	Yes	Lung contusion (18) rib fracture (8), hemo-pneumothorax (6), cardiac injury (3) sternal fracture (1), scapula fracture (1), vascular injury (5), esophageal injury (1)	1 Death
**Hiss J**. **1997**	Rubber and steel/15.4 g/100 m/s/41.5 J and Plastic 0.85 g/1225 m/s/663.7 J	*	17/2	Yes	Lung and heart lacerations	2 Deaths
**Voiglio E.J** **1998**	Rubber pellets/*/*	Contact	1/1	Yes	Hemothorax, rib fracture, cardiac laceration.	Died
**Chute DJ** **1998**	Plastic 79.4 g/74 m/s/220 J	*	1/1	No	Hemothorax, rib fracture, lung laceration, cardiac laceration	Died
**Steele J.A** **1999**	Plastic 135 g/70 m/s/332 J	*	155/25	*	*	All survived
**Mahajna A**. **2002**	Rubber 48 g/130 m/s/46 J and 17 g/78 m/s/33 J	30–80	152/39	Yes	Lung contusion and rib fracture (8), pneumothorax (6), hemothorax (4), cardiac tamponade (1), cardiac contusion (1), vascular injury (1)	All survived
**Kalebi A**. **2005**	Rubber pellets */*/*	*	1/1	Yes	Hemothorax, lung laceration, rib fracture	Died
**Hughes D**. **2005**	Plastic 98 g/64 m/s/244 J	*	28/7	No	Lung contusion	All survived
**Wahl P**. **2006**	Rubber 28 g/*/200 J	2	2/1	No	Lung contusion, cardiac contusion	Survived
**Maguire K. 2007**	Plastic attenuated energy 28 g/*/200 J	*	13/2	No	Pneumothorax (1)	Survived
**Chowaniec C. 2008**	Rubber 8 g/94 m/s/40 J and pellets 0.3 g/215 m/s/7.3 J	*	1/1	Yes	Hemothorax, lung laceration, cardiac laceration	Died
**Rezende-Neto J. 2009**	Rubber attenuated energy 19 g/130 m/s/ 200 J	2	1/1	Yes	Pneumothorax, lung laceration	Survived

Range in meters; * Missing information; ^children; ^• ^only patients with penetrating chest injuries were included in the study.

When a projectile strikes a person, its kinetic energy at impact is defined by its mass and its velocity (1/2 × mass × velocity^2^). Ballistic studies suggest that a projectile needs to apply a "threshold energy density" of greater than 0.1 J/mm^2 ^to skin in order to penetrate and cause internal injuries [5]. Manufacturers of rubber bullets modify the composition (mass: rubber vs lead), ballistic properties (velocity) and size (cross-sectional area) in order to reduce the likelihood of skin penetration. Furthermore, law-enforcement officers often have specific "rules of engagement" for using these types of munitions that further reduce the likelihood of penetration and serious injury; such rules include firing at distances over 40 meters and changing the point of aim to body regions where skin has increased elastic properties (lower anterior abdomen or thigh) to allow the energy to dissipate over a larger cross-sectional area [6].

One broad classification of "less lethal" impact munitions is direct versus indirect fire rounds. Indirect fire munitions are made of relatively dense material, and are therefore fired in front of targets with the purpose of "skipping" them into targets. Accuracy, however, is lost and the chance of hitting "non-elastic" structures such as the head and the chest increases, and therefore, causing greater risk of serious injury or death [7].

Direct-fire rubber bullets were used for the first time by British Forces in Northern Ireland in 1970 [8]. These bullets were also relatively inaccurate, as such, many injuries and even some deaths were associated with their use [3,8,9]. Children, teenagers, and women who are of a smaller built were reported to sustain severe injuries more often than larger individuals, particularly to the skull, eyes, brain, lungs liver, and spleen. [3,9-11]. That is in keeping with the results of a previous study, performed on unembalmed cadavers, that demonstrated greater injury risk of blunt ballistic impacts in 5^th ^percentile female patients – abbreviated injury severity score chest (AIS-chest 1) – compared to 50^th ^percentile males (AIS-chest 2) struck by a 12-gauge rubber bullet with a mass of 6 g fired at a velocity of 122 m/s [12]. Furthermore, injury tolerance curves showed that if the mass of the bullet is increased to 140 g the velocity should be reduced to 18 m/s to avoid serious injuries to the chest of a female; a speed that is well below that of current "less-lethal" munitions [12].

Because of these safety concerns, rubber bullets have been replaced by plastic rounds in many countries [1-3]. The latter are more accurate and have less wounding potential [1,3,6,8]. Interestingly however, the reported fatality rate of plastic bullets is approximately 1:4000 bullets fired as opposed to 1:18000 for rubber bullets. Those numbers, however, may be misleading because of the many different projectiles with variable wounding power used around the world [6,8,10,11]. Nonetheless, similar to rubber bullets, the head and the chest are arguably the areas of the body most vulnerable to severe injuries caused by plastic rounds [2,3,10,11,13].

Out of the 18 articles reviewed in this study plastic bullets were used in 11, while rubber bullets were used in 8 others; one study reported both types of ammunition. There were 4 deaths from intra-thoracic injuries caused by rubber bullets and 8 deaths from intra-thoracic injuries provoked by plastic ones [11,13-17].

With respect to intra-thoracic penetration, it was recently demonstrated in post-mortem human subjects, using a 12-gauge (6.4 g) rubber bullet, that the region with lowest average energy for penetration impact was the area between the ribs (33.1 J/cm^2^), while the posterior rib area had the highest energy density for penetrating events (55.9 J/cm^2^) [18]. Thus, based on our review, many "less-lethal" munitions have impact energy above the threshold for penetration; including the one described in the present case report (200 J). Therefore, it is not surprising that intra-thoracic penetration was described in more than half of the reports that were reviewed [6,13,14,16,17,19-22]. It is interesting to note that significant injuries, such as, rib fractures, pneumothorax, hemothorax, and contusions to the heart and lung also occurred independently of intra-thoracic penetration; including the death of a female patient who sustained left ventricle and pulmonary lacerations [1-3,8,9,11,23,24].

In pursue of safer "less-lethal" impact munitions manufactures developed the attenuated energy projectiles (AEP). These bullets were designed to duplicate the ballistic performance of the advanced plastic baton rounds but reduce the risk of serious injury in cases of inaccurate fire [2]. These types of projectiles have a deformable head above the solid polyurethane polymer base of the standard plastic baton rounds [25]. On inadvertently hitting a hard target like the head or the chest, the AEP should deform, spreading the impact over a greater area and a longer time period, decreasing the likely hood of serious injury and penetration. Furthermore, they provide better firing accuracy than previous plastic bullets, and do not fragment reducing the risk of accidental injuries [2]. However, a recent report of 13 patients demonstrated that even attenuated energy projectiles are associated with a 37% incidence of significant injuries to the head, neck, and the chest (AIS 2–5), but there were no cases of intra-thoracic penetrating [2]. Our case apparently is the first one in which there was intra-thoracic penetration by an attenuated energy projectile.

In summary, to decrease serious injury caused by "less-lethal" impact munitions, the "rules of engagement" should be rigorously followed, even if the munition is an AEP.

## Conclusion

Even though the nature of the wound caused by attenuated energy bullets is generally blunt, penetration can occur specially when fired from close range at the torso. Therefore, patients who sustain less lethal ammunition injury to the chest should be thoroughly investigated with chest radiography and CT scan regardless of the ballistic features of the projectile.

## Competing interests

The authors declare that they have no competing interests.

## Authors' contributions

JBRN drafted the manuscript and operated on the patient. FDFS, LBOP, and LCT have been involved in drafting the manuscript and the operation; HT, expert opinion on ballistics and revising the manuscript for important intellectual content; SBR, drafting and revising the manuscript for important intellectual content; All authors gave final approval of the version to be published.

## Consent

A written informed consent was obtained from the patient for publication of this case report and any accompanying images. A copy of the written consent is available for review by the Editor-in-Chief of this journal.
